# Ray-finned fishes (Actinopterygii) from the Upper Jurassic (Oxfordian) of the Atacama Desert, Northern Chile

**DOI:** 10.7717/peerj.13739

**Published:** 2022-08-02

**Authors:** Rodrigo A. Otero

**Affiliations:** 1 Laboratorio de Ontogenia y Filogenia, Departamento de Biología, Facultad de Ciencias, Universidad de Chile, Ñuñoa, RM, Chile; 2 Paleo Consultores, Santiago, RM, Chile; 3 Museo de Historia Natural y Cultural del Desierto de Atacama, Interior Parque El Loa s/n, Calama, Región de Antofagasta, Chile

**Keywords:** Actinopterygians, Upper Jurassic, Paleobiogeography, Taxonomy, Southeastern Pacific

## Abstract

This contribution presents novel records of ray-finned fishes from the Oxfordian of Cerritos Bayos, northern Chile. This includes a Pachycormiformes diversity represented by macropredatory forms (aff. *Hypsocormus* sp. and a still indeterminate form) and by suspension-feeding forms (*Leedsichthys* sp). The assemblage also includes the first Upper Jurassic local record of a Lepisosteidae, the latter being the oldest known to date in Gondwana. This diversity is complemented by new material of the lepidotid genus *Scheenstia*. The ray-finned fish assemblage from the Oxfordian of Cerritos Bayos is dominated by Lepisosteiformes and Pachycormiformes, complementing previous local coeval records from El Profeta Formation (ca. 250 km south from the localities here studied), mostly comprised by small Teleostei (*e.g*., *Protoclupea chilensis*, *Varasichthys ariasi*, *Chongichthys dentatus*, among others), indeterminate Pachycormiformes and Pycnodontiformes (*Gyrodus* sp.). The new records extend the known actinopterygian diversity from the Upper Jurassic of southeastern Panthalassa.

## Introduction

Jurassic ray-finned fishes (Osteichthyes) from northern Chile are among the best documented fossil vertebrates in the country. In particular, the local Upper Jurassic record includes several stem-Teleostei endemic species (Arratia, 1981, 1982, 1986, 1987; Arratia & Schultze, 1985, 1987, 1999; Arratia, Chang & Chong, 1975a, 1975b, 1975c) and a few genera with indeterminate species (Biese, 1961; Arratia & Cione, 1996; Martill, Chong & Pardo, 1998; Martill et al., 1999; Kriwet, 2001; Liston, 2008, 2010; Ossa-Fuentes et al., 2015). Most of these taxa were recovered from Oxfordian levels of the El Profeta Formation (Chong, 1973), with only few coeval specimens having being described elsewhere in the northern part of the country. This contribution presents material collected in the last years from Oxfordian beds exposed west of Calama, ca. 250 km north from the outcrops of the El Profeta Formation. The material provides evidence of a pachycormid diversity, filling the austral gap during the Oxfordian. Together with these, the same study area provided the first local Upper Jurassic record of a lepisosteid, in association with material referable to the genus *Scheenstia*. This assemblage contrasts with the rich assemblage of small Teleostei described from Oxfordian levels of the El Profeta Formation, 250 km south in the same Atacama Desert of northern Chile. Their implications are here discussed.

### Locality and geological setting

**Figure 1:**
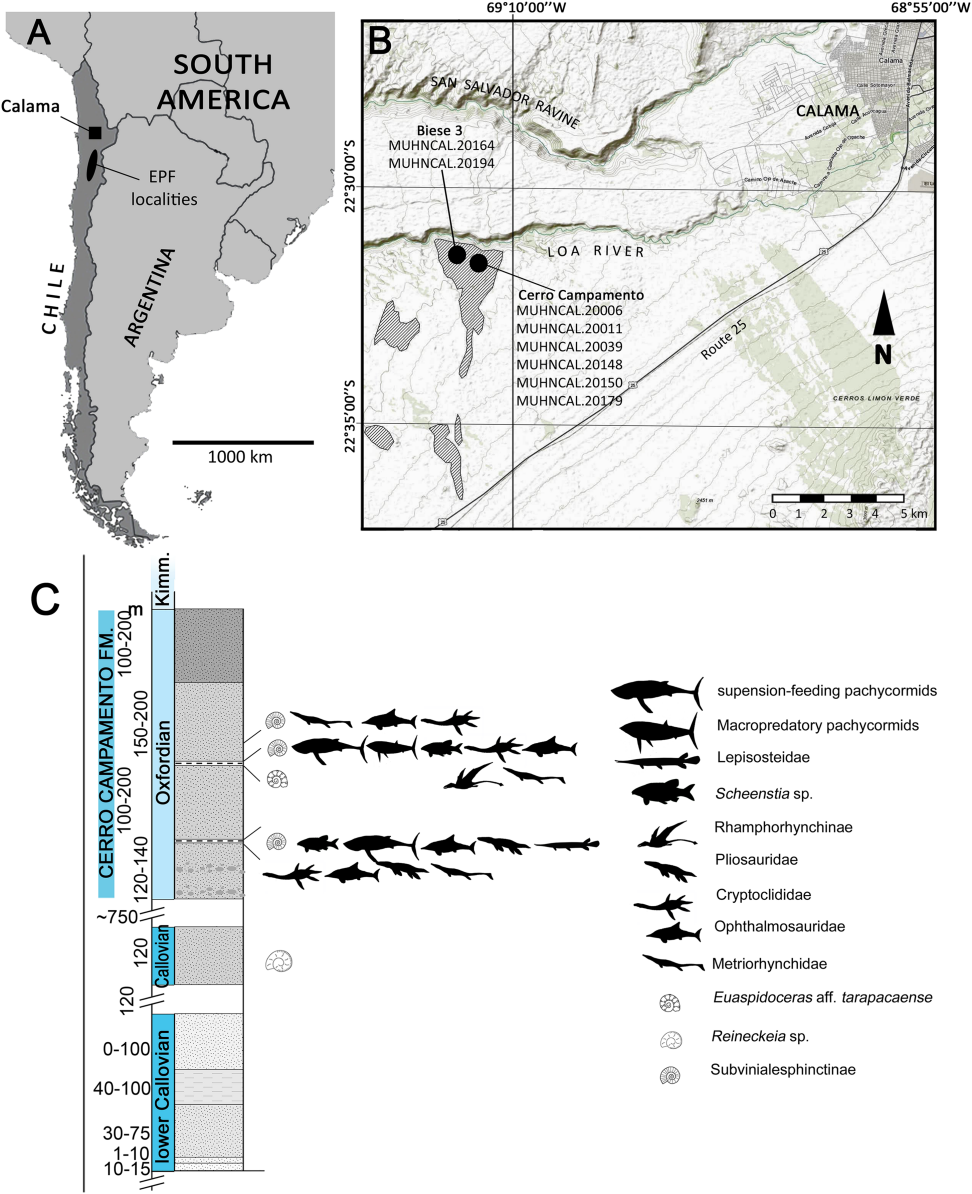
Locality and stratigraphic provenance. (A) Map indicating the Upper Jurassic outcrops and the localities where the material was recovered. Outcrops of the El Profeta Formation (EPF) are indicated for reference. (B) General stratigraphic section of the Cerro Campamento Formation, indicating the relative stratigraphic position of the different taxa recovered. Map from World Topo Map (Open Source: http://www.andeangeology.cl/index.php/revista1/article/view/V2n1-a02). Distribution of outcrops based on Biese (1961) and Lira (1989). Thickness data updated after Duhart et al. (2018).

The material studied here was recovered from two localities near Calama, northern Chile (Fig. 1). Most of the specimens were found in Cerro Campamento, 30 km west of Calama, Región de Antofagasta. A single specimen was recovered from a different locality, informally named Biese 3, 400 m south from Loa River. The fossil-bearing levels at Cerro Campamento are part of the Cerro Campamento Formation (Lira, 1989; Duhart et al., 2018). This unit was first considered to be a member of the former Cerritos Bayos Formation (Biese, 1961; Lira, 1989), and later reassessed as a proper formation by Duhart et al. (2018). It is mostly comprised of well-stratified, calcareous black limestones, fine calcareous sandstones, fossiliferous shales and gypsum levels and dykes, with an estimated thickness of 450 m. Its age is constrained between the ?Bathonian and the Kimmeridgian, mostly based on ammonoid biostratigraphic correlations (Duhart et al., 2018). The associated presence of Vinalesphinctinae taxa (*e.g*., *Cubasphinctes*, *Subvinialesphinctes*: see Parent, 2006) together with individuals referable to *Euaspidoceras* sp., suggest a middle-to-upper Oxfordian age for the Cerro Campamento strata.

The outcrops of the Biese 3 locality also correlates with the Cerro Campamento Formation. The presence of *Reineckeia* sp. slightly below the fossil-bearing strata in the studied section, plus the direct association of Vinalesphinctinae taxa (likely *Cubasphinctes*) suggests a local lower Oxfordian age for the fossil-bearing level.

## Materials and Methods

Most the studied material was recovered as scattered elements already separated from their lithostratigraphic context, but close to their natural occurrences. This observation is based on the sharp edges of the blocks and the possibility of reassembling them, which suggests limited transportation from their *in situ* position. MUHNCAL.20006, MUHNCAL.20148 and MUHNCAL.20039, are blocks with bones exposed by natural cracking. These specimens have only been consolidated with cyanocrylate, but further preparation was not undertaken because most of the bony material is exposed as a thin patina. MUHNCAL.20150 was found scattered in several fragments already separated from the matrix, and later reconstructed in facilities of the Museo de Historia Natural y Cultural del Desierto de Atacama, Calama, Chile (Museum of Natural and Cultural History of the Atacama Desert), being consolidated with cyanocrylate. MUHNCAL.20164 is a skull contained in a naturally fragmented concretion that permits observation of several very delicate bony elements. Due to this, its current preliminary description considers only the exposed surfaces. The scales of MUHNCAL.20011 were mechanically prepared with hand tools, due to the easily removable sediment covering it. MUHNCAL.20193 and MUHNCAL.20194 were prepared with an electric engraver and PaleoTools 9100 air scribe in the facilities of the Laboratorio de Ontogenia y Filogenia, Departamento de Biología, Universidad de Chile (Santiago, Chile).

Anatomical terminology of the branchial elements follows Liston (2013). Traditional terminology of the caudal anatomy follows Arratia & Lambers (1996). Anatomy of lepisosteids is slightly modified from Grande (2010), adding indications of the laterality in each case.

**Permissions—**Retrieval of fossil specimens in Chilean territory was granted by Consejo de Monumentos Nacionales (National Monuments Council) in Ord. CMN N°1464/2021.

**Systematic Paleontology**

ACTINOPTERYGII Cope, 1887

NEOPTERYGII Regan, 1923

PACHYCORMIFORMES Berg, 1937

PACHYCORMIFORMES INDET.

(Fig. 2)

**Figure 2:**
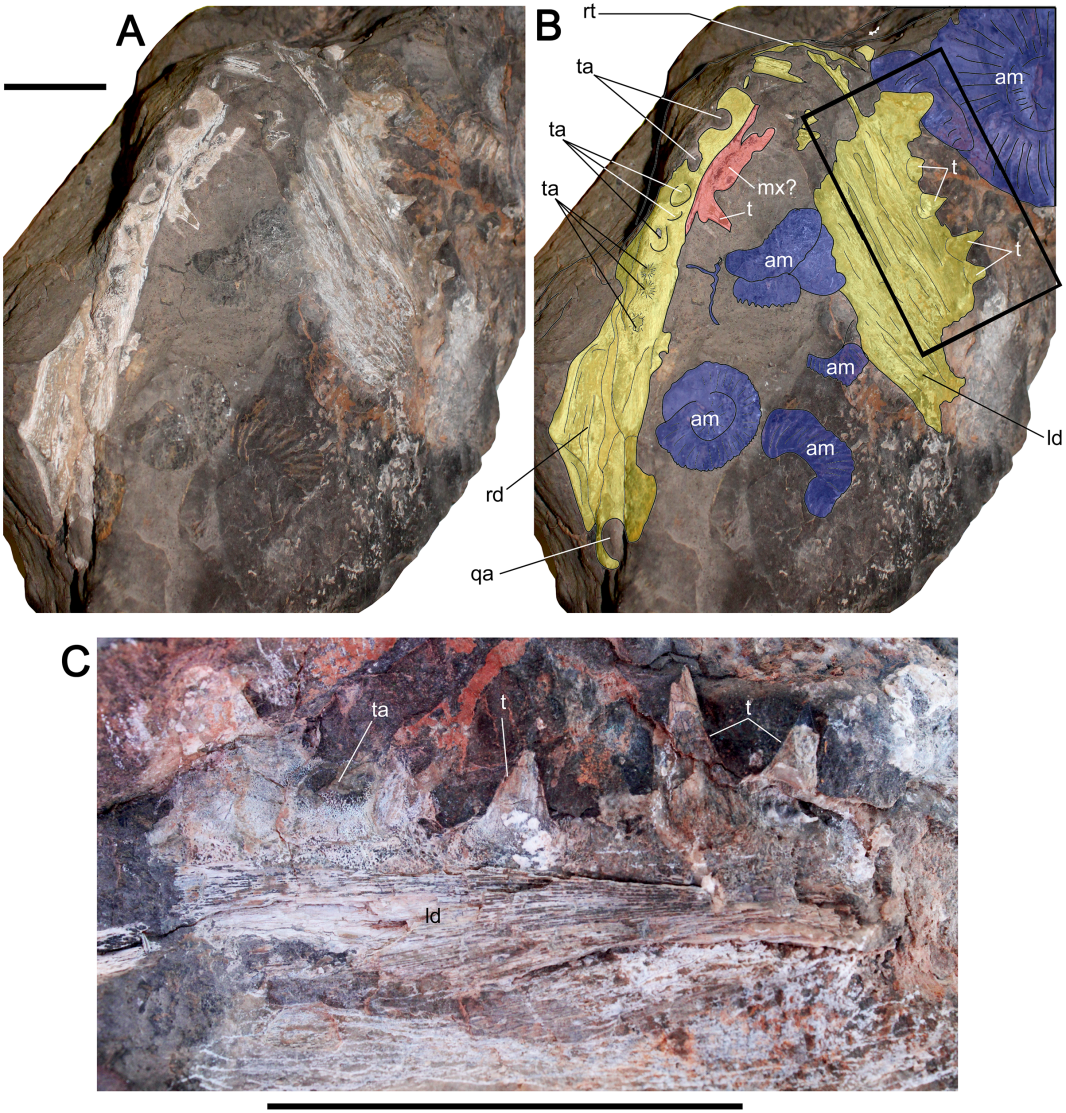
Macropredatory Pachycormiformes from Cerro Campamento Formation, northern Chile. Pachycormiformes indet. MUHNCAL.20039. Incomplete jaw. Cerro Campamento, west Calama, middle to upper Oxfordian. (A) Ventral view of the partial mandible, showing the right dentary in internal view and the left dentary in lateral view. (B) Anatomical interpretation of the previous. (C) Detail of the dentition (square in B), showing the broad, conical and non-procumbent teeth. Anatomical abbreviations: am, ammonoids (Vinialesphinctinae); ld, left dentary; mc, meckelian canal; mx?, maxillary? fragment; qa, quadrate articulation; rd, right dentary; rt, right dentary; t, tooth; ta, tooth attachment. Scale bar equals 5 cm in all cases.

aff. *Eugnathides*: In Ossa-Fuentes et al., 2015.

**Material—**MUHNCAL.20039, a fragmentary mandible preserving parts of both dentaries. Cerro Campamento, west Calama. Cerro Campamento Formation, middle-to-upper Oxfordian.

**Description—**The mandible is crushed. The right ramus is preserved in occlusal view and the left one in external view (Figs. 2A, 2B). The left ramus preserves the incomplete dentary, which has its external surface eroded, but part of its ventral margin is preserved, allowing the conclusion that the mandible was dorsoventrally high. The right dentary is fragmentary, being exposed in occlusal view. The latter preserves at least seven tooth attachments visible on cross-section. Its posterior margin is broken, exposing an oval facet in cross-section, consistent with the quadrate articulation. A small bony fragment is crushed and contacts the right dentary along the occlusal margin. This bony element preserves a fragmentary tooth, which is oriented in the opposite direction to those of the dentary, thus suggesting that this belongs to the complementary occlusal element, for which reason it is doubtfully referred to a maxillary fragment (it could be also a premaxillary fragment). The left dentary preserves its occlusal margin with four poorly preserved teeth. The preserved portion of teeth is robust and straight, with a triangular crown outline in lateral view. Based on the observable alveolar shape (Fig. 2C), each crown has a nearly rounded cross-section, suggesting that the crowns are cylindrical. The teeth are strongly fused to the bony tissue of the dentary.

**Remarks—**MUHNCAL.20039 has large teeth almost vertically directed, with conical and relatively blunt crowns. Similar features have been described for pachycormids, such as *Simocormus macrolepidotus* (Maxwell et al., 2020: fig. 3). Coeval ray-finned fishes with similar dentition and relative size includes the Amiiformes genus *Caturus* (see Tyborowski, 2017: fig. 2). The latter differs by having teeth apically recurved, while those of MUHNCAL.20039 are straight. Moreover, Amiiformes possesses several rows of coronoid and prearticular teeth medial to the dentary teeth. These are less than half the height of the dentary teeth and are rounded at the tip (Grande & Bemis, 1998: p. 84). These coronoid and prearticular tooth rows are absent in MUHNCAL.20039. For the moment, MUHNCAL.20039 is kept as an indeterminate Pachycormiformes awaiting more complete material.

PACHYCORMIDAE Woodward, 1895

Genus *HYPSOCORMUS* Wagner, 1863

AFF. *HYPSOCORMUS* SP.

(Figs. 3A–3F)

**Figure 3:**
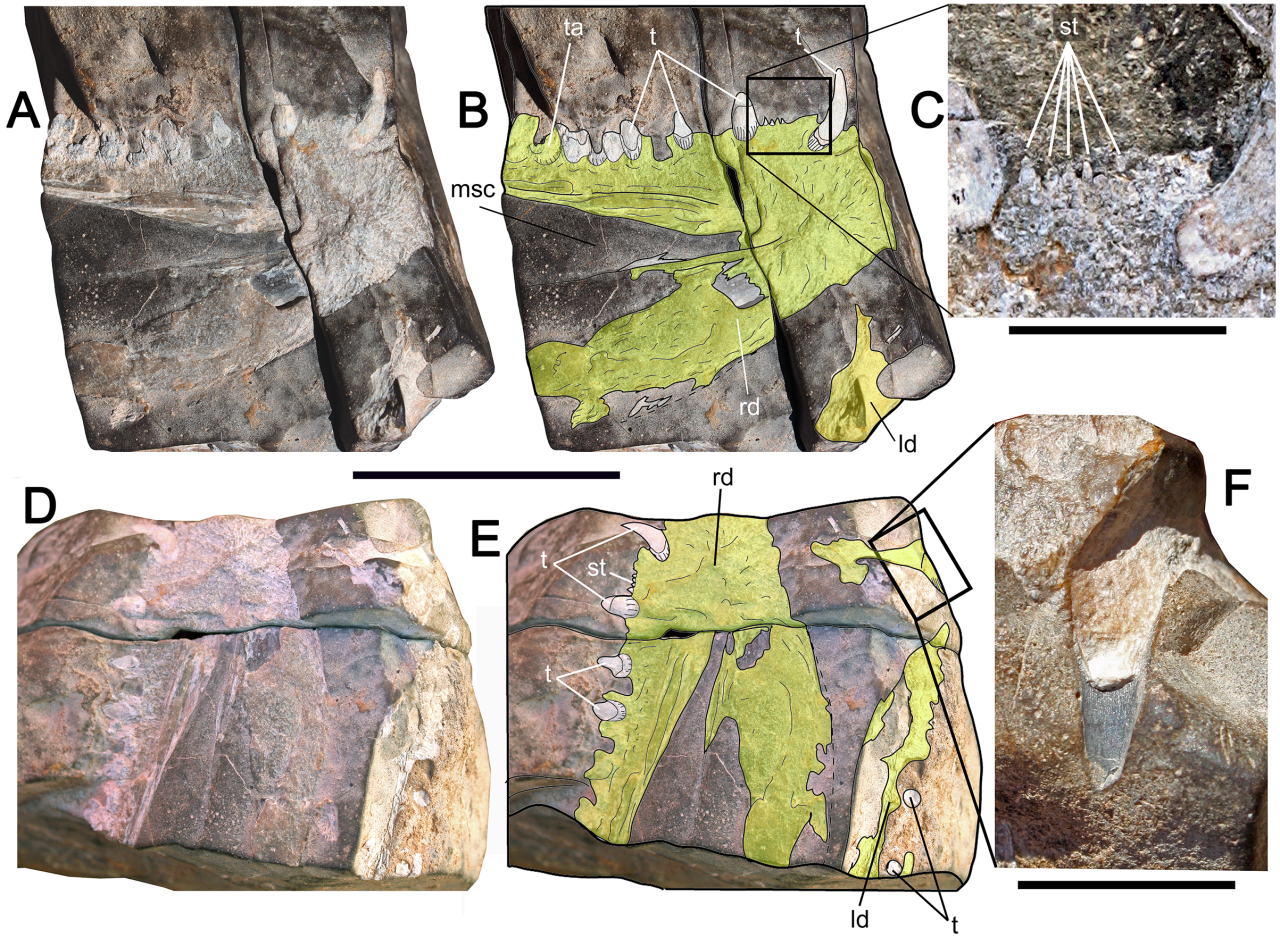
Macropredatory pachycormids from Cerro Campamento Formation, northern Chile. aff. *Hypsocormus* sp.; MUHNCAL.20148. Incomplete jaw. Cerro Campamento, west Calama, middle to upper Oxfordian. (A) Right dentary in right view. (B) Anatomical interpretation of the previous. (C) Close-up of the square area, showing the small occlusal denticles. (D) ventral view. (E) Anatomical interpretation of the previous. (F) Close-up of the square area, showing the details of one anterior main tooth. Anatomical Abbreviations: mc, mandibular canal; ld, left dentary; st, small teeth; rd, right dentary; t, teeth; ta, tooth attachment. Scale bar equals 50 mm except C and F, equals 10 mm.

Pachycormiformes: In Alarcón-Muñoz et al., 2020, 2021.

**Type Species—***Hypsocormus insignis* (Wagner, 1860), Late Jurassic, Bavaria, Germany.

**Material—**MUHNCAL.20148, part of both mandibular rami. Cerro Campamento, west Calama. Cerro Campamento Formation, middle-to-upper Oxfordian.

**Description—**MUHNCAL.20148 preserves both mandibular rami partially displaced from each other, with their respective posterior ends missing. The right dentary is the best preserved (Figs. 3A, 3B). This shows at least five main teeth and three gaps corresponding to missing dental attachments. Over the occlusal margin, very small teeth are present (Fig. 3C). Due to the nature of its preservation, it is hard to assess if the small teeth are part of the dentary or the coronoid. The external dentary wall is mostly eroded, however, its ventral part is preserved, as well as the internal mould of the dentary impressed onto the matrix (Figs. 3D, 3E). This allows its laterally triangular and dorsoventrally deep outline to be assessed. The mandibular sensory canal is exposed. A small section of the dentary symphysis is lost. The mold of an anterior tooth is preserved in the left dentary (Fig. 3F). This shows profuse and fine striations which seems to fade apically (the tip is broken).

**Remarks—**MUHNCAL.20148 conforms to the dentary of a macropredatory pachycormid, being in the size range of *Orthocormus*, *Simocormus* and *Hypsocormus* (Arratia & Schultze, 2013; Maxwell et al., 2020). It differs from *Simocormus* in the comparatively larger denticles of the latter, its dentary ornamentation and by its conical (not procumbent) dentary teeth (Maxwell et al., 2020: fig. 3). The dentary of *Orthocormus* is characterized by the presence of large procumbent conical teeth, the lack of small lateral teeth and three large teeth anteriorly (Lambers, 1988: plate 2A; Arratia & Schultze, 2013: fig. 5A; Martill & Brito, 2020: p. 56). Such features are clearly different from MUHNCAL.20148. The Chilean specimen shares with *Hypsocormus* and the Albian *Australopachycormus* the presence of large conical teeth with numerous small lateral teeth between (Kear, 2007; Arratia & Schultze, 2013: p. 93).

The genus *Hypsocormus* is restricted to the Callovian-lower Tithonian of Europe (Arratia & Schultze, 2013; Maxwell et al., 2020; Martill & Brito, 2020); it is also present in Cuba with records of Oxfordian (‘*Sauropsis*’ in Gregory, 1923; Iturralde-Vinent & Ceballos, 2015; Maxwell et al., 2020) and Tithonian age (Iturralde-Vinent & Ceballos, 2015). Based on the morphological affinities and the chronostratigraphic occurrence of MUHNCAL.20148, the latter is referred to as aff. *Hypsocormus* sp.

Genus *LEEDSICHTHYS* Woodward, 1889

*LEEDSICHTHYS* SP.

(Figs. 4 and 5)

**Figure 4:**
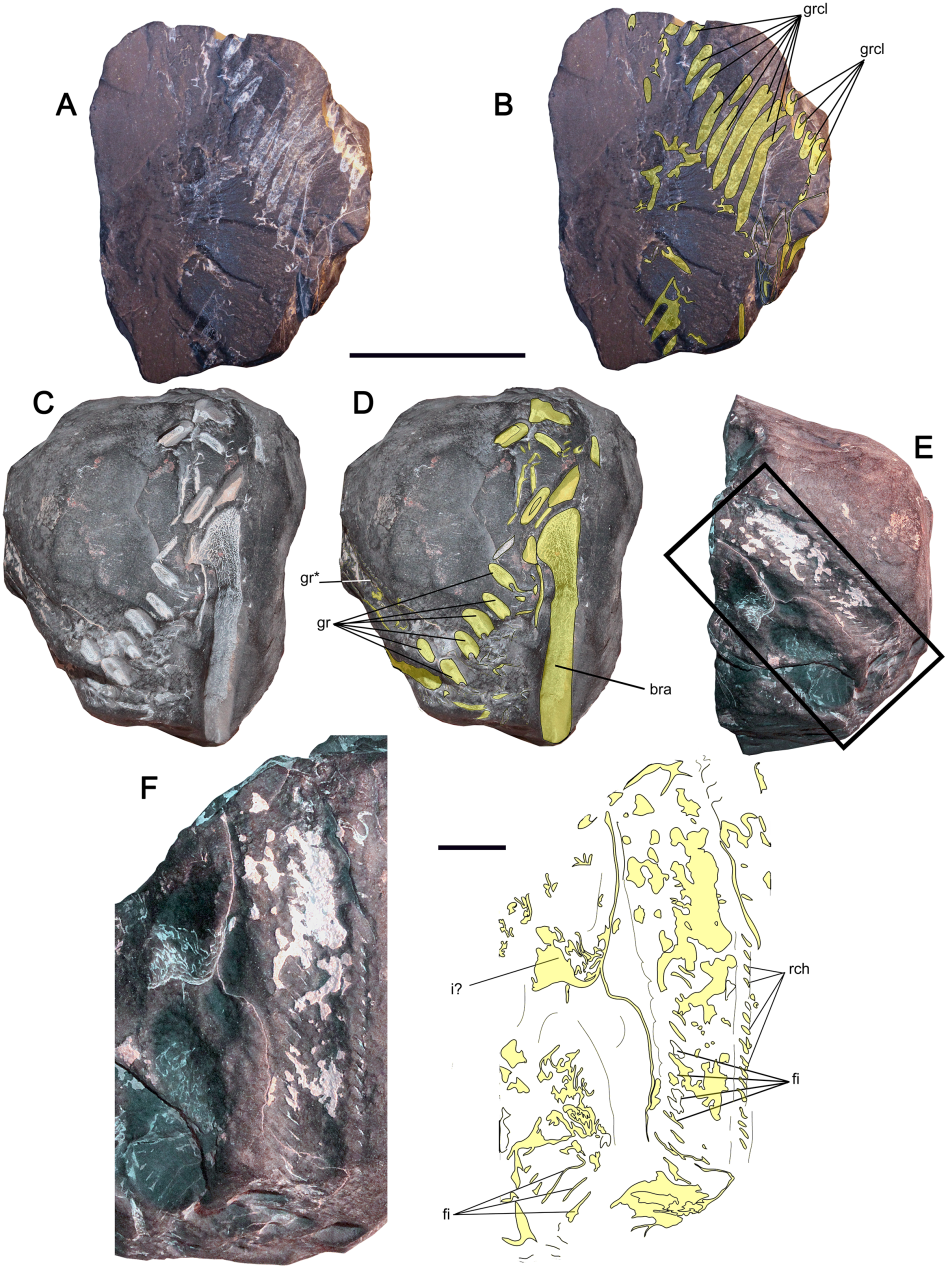
Suspension-feeding pachycormids from Cerro Campamento Formation, northern Chile. *Leedsichthys* sp.; MUHNCAL.20006. Block preserving at least very large gill rakers. Cerro Campamento, west Calama, middle to upper Oxfordian. (A) Side of the block showing the larger cross-section of the gill raker cluster in distal view. (B) Anatomical interpretation of the previous view. (C) Side of the block showing one gill filament in longitudinal section. (D) Opposite view of A, showing the proximal cross-section of the gill raker cluster. (E) Longitudinal section of a gill filament (squared area marked on C). (F) Anatomical interpretation of the previous. Anatomical abbreviations: bra, branchial arch fragment; fi, fimbriations; gr, gill raker; grcl, gill raker cluster; rch, resorption chambers. Scale bar equals 50 mm.

**Figure 5:**
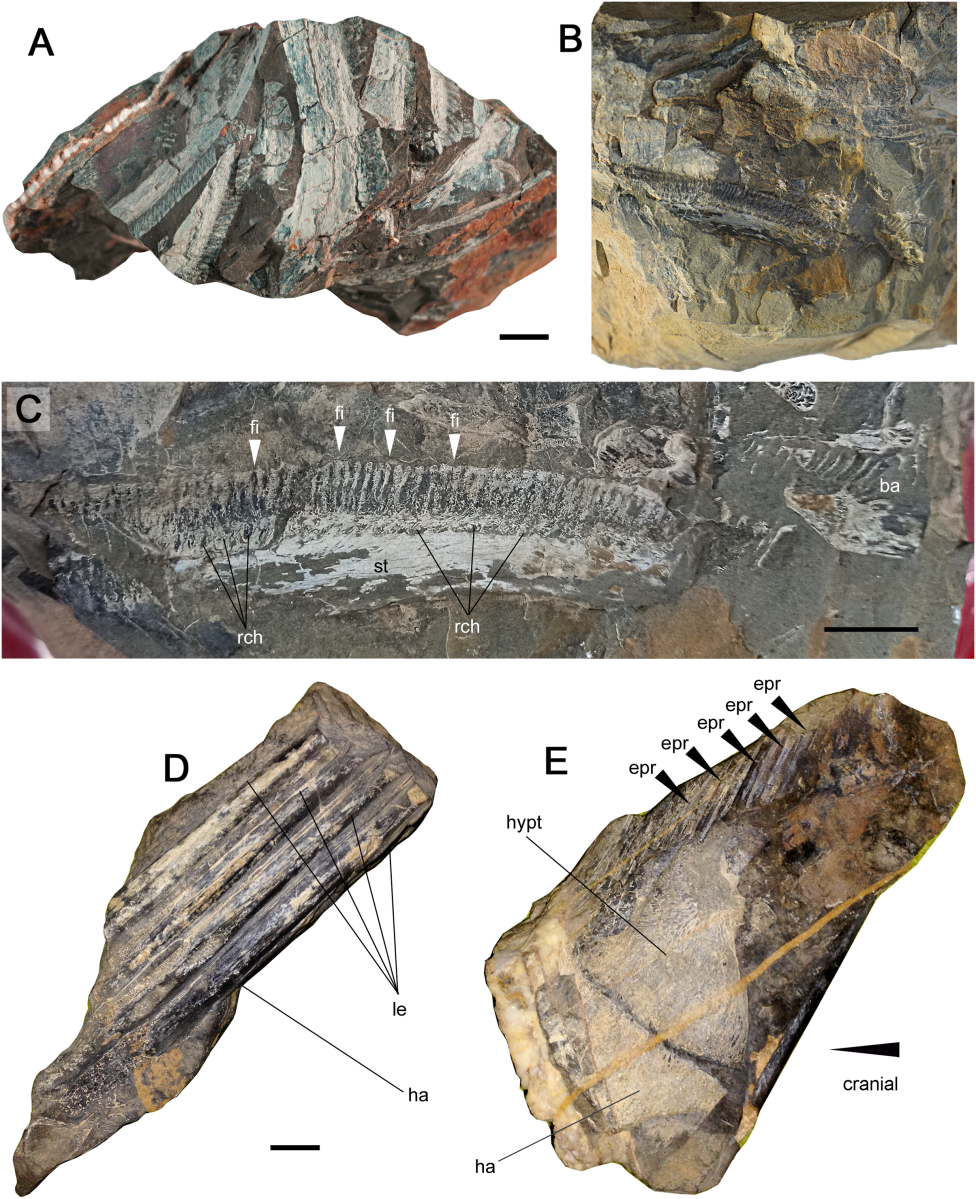
Suspension-feeding pachycormids from Cerro Campamento Formation, northern Chile. *Leedsichthys* sp. MUHNCAL.20226, broken concretion preserving several gill rakers. Cerro Campamento, west Calama, middle to upper Oxfordian. (A) block with at least eight gill rakers. (B) opposite block preserving two gill rakers. (C) close-up of the previous, showing profuse fimbriations and few resorption chambers in the stalk. MUHNCAL.20150, fragment of a caudal fin. (D) View of the parallel principal rays. (E) Opposite view of the same block. Anatomical abbreviations: ba, base; epr, epaxial rays; fi, fimbriations; ha, caudalmost haemal arch; hypt, hypural plate; le, lepidotrichia; st, stalk; rch, resorption chambers. Scale bar equals 1 cm in all cases.

**Type Species—***Leedsichthys problematicus* Woodward, 1889. Callovian of the Oxford Clay, UK and France.

**Material—**MUHNCAL.20006, a block preserving at least twelve gill rakers. MUHNCAL.20226, an associated group of gill rakers. MUHNCAL.20150, a fragment of a caudal fin. Cerro Campamento, west Calama. Cerro Campamento Formation, middle-to-upper Oxfordian.

**Description—**MUHNCAL.20006 is a compact shale block that internally hosts several flattened structures. On one side, at least eight, flattened structures are ordered in parallel (being 7.5 cm their preserved length), plus a second cluster with four smaller structures (Figs. 4A, 4B). On the opposite side of the same block, at least 13 smaller structures (less than 1 cm) are visible, with six of them in parallel order, while part of these are slightly scattered (Figs. 4C, 4D). Orthogonal to the latter view (Figs. 4E, 4F), one of these structures is visible in longitudinal section due to erosion. This shows a profuse and ordered pattern of parallel, delicate bony structures representing the oblique edges of two gill raker stalks, along with strands and fragments of the suprafanuncular mesh (Liston, 2008, 2013).

MUHNCAL.20226 is preserved in a single concretion which was naturally broken into two blocks. One block shows at least eight, well-preserved gill rakers (Fig. 5A), while two of them are preserved in the opposite block (Fig. 5B). The gill rakers are all incomplete, with their distal ends missing. These have profuse fimbriations over the stalk, and in a few cases, the exposed resorption chambers in the stalk (Liston, 2013; Gouiric-Cavalli, 2017) can be observed below the fimbriations (Fig. 5C).

MUHNCAL.20150 preserves four principal rays (each one having ca. 10 mm width; 11.1 cm the longest preserved fragment). These appear as tabular parallel structures without evidence of segmentation. On the opposite side of the rays, two massive bone fragments are visible, being interpreted as the hypural plate and a fragment of the caudalmost haemal arch. Ten small parallel bony rays lie laterally adjacent to the larger bones, being interpreted as epaxial rays.

**Remarks—**The preserved gill rakers are ca. 7 cm long. Their missing distal parts are estimated to be no more than 3 cm, based on the distal reduction of the rakers observed in cross-section. This indicates that the preserved fragment represents a portion near the tip of the raker stalk. The length of the missing proximal part is unknown, although this should largely be exceeded by the length of the preserved portion.

MUHNCAL.20226 gill rakers are almost identical to the material illustrated by Liston (2010: fig. 11; 2013: fig.1F, 7, 8) and Gouiric-Cavalli (2017), both referred to the genus *Leedsichthys*. In addition, the gill raker sizes of MUHNCAL.20006 are coincident with those of MUHNCAL.20226, a further reason why both were identified as *Leedsichthys* sp. In Gondwana, the genus *Leedsichthys* has been previously recorded in the Oxfordian of northern Chile (Martill et al., 1999) and in the lower Tithonian of Argentina (Gouiric-Cavalli et al., 2019).

The presence of unsegmented rays in MUHNCAL.20150, plus the epaxial rays in their anatomical position, indicate that this is a proximal fragment of a very large caudal fin (Liston et al., 2013), while the bony elements array is similar to that illustrated by Arratia & Schultze (2013: fig. 9). Moreover, the lack of segmentation is considered to be a distinctive feature of the caudal fins of suspension-feeding pachycormids (Friedman et al., 2010; Arratia & Schultze, 2013; Liston et al., 2013; Cione et al., 2018). The very large size of MUHNCAL.20150 rays are remarkably similar to those of the caudal specimen NHMUK.PV.10000 referred by Liston et al. (2013) to *Leedsichthys problematicus*, although their antero-posterior width is comparatively smaller than those of the latter specimen (15 mm width). The lack of more complete remains, however, prevents a definitive specific referral.

HOLOSTEI Müller, 1844 (*sensu* Grande, 2010)

GINGLYMODI Cope, 1872 (*sensu* Grande, 2010)

LEPISOSTEIFORMES Hay, 1929 (*sensu* López-Arbarello, 2012)

LEPISOTEOIDEA López-Arbarello, 2012

LEPISOSTEIDAE Cuvier, 1825

LEPISOSTEIDAE INDET.

(Figs. 6A–6C)

**Figure 6:**
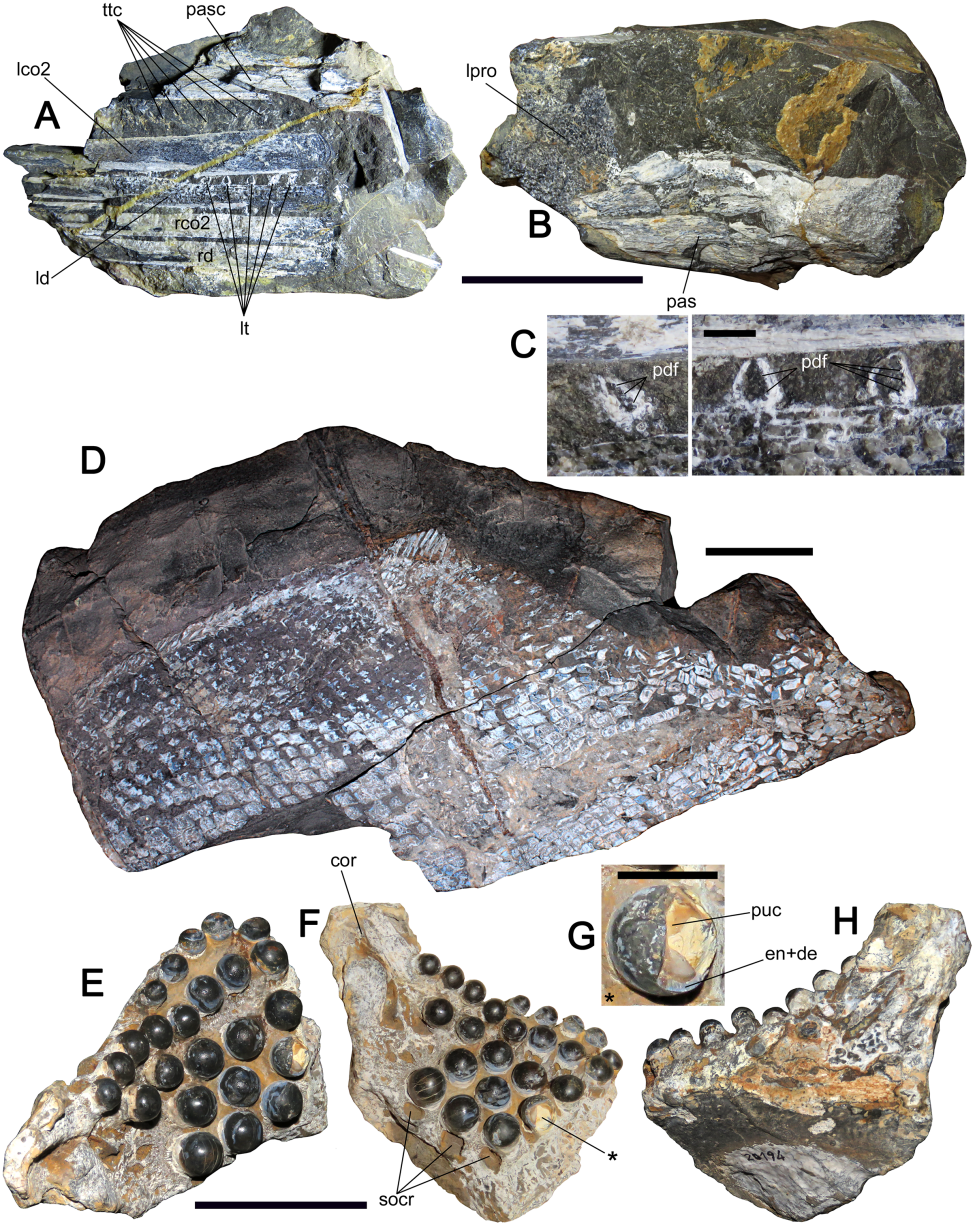
Lepisosteids and lepidotids from Cerro Campamento Formation, northern Chile. (A) Lepisosteidae indet.; MUHNCAL.20164, fragmentary skull. Biese 3 informal locality, west Calama, lower Oxfordian. Laterally crushed mandibular rami in left view. (B) Left view of the basisphenoid and part of the left prootic. (C) Close-up of the large teeth in oblique cross-section, showing the internal folds of the plicidentine. (D) *Scheenstia* sp.; MUHNCAL.20011, articulated body scales and dorsal fin in left lateral view. Cerro Campamento, west Calama, middle to upper Oxfordian. (E) *Scheenstia* sp.; MUHNCAL.20194, left dentary in occlusal view. Biese 3 informal locality, west Calama, lower Oxfordian. (F) Same in lingual view. (G) Close-up of a medial broken crown, indicated with an aterisk on F. (H) Left labial view. Anatomical Abbreviations: cor, coronoid process; en+de, enamel+dentine; lco2, left coronoid 2; ld, left dentary; lpro, left prootic; lt, large teeth; pas, parasphenoid; pasc, parasphenoid cast; pdf, plicidentine folds; puc, pulp cavity; rco2, right coronoid 2; rd, right dentary; socr, semi-open bony crypts; ttc, tiny teeth cluster. Scale bar equals 5 cm except in C, 3 mm and G, 10 mm.

**Material—**MUHNCAL.20164, a single cracked block preserving a fragmentary skull which includes part of the braincase, fragments of both mandibular rami, and part of the posterior dermal skull. Informal locality named Biese 3, 400 m south of Loa River, west Calama. Cerro Campamento Formation, lower Oxfordian.

**Description—**The material is preserved in a single concretion naturally fractured into three blocks. The braincase is three-dimensionally preserved, including most of the parasphenoid and both incomplete prootics. Sections of both mandibular rami are preserved. The left dentary is exposed in inferior oblique view and preserved as counterparts. These show at least seven large teeth with tiny teeth on the crown base. In cross-section, the large teeth show internal folds of the dentine (plicidentine in Grande, 2010). Parallel to the dentary lies a similar bone, here interpreted as the left coronoid 2, which displays at least nine clusters of tiny teeth. The dentary and the coronoid 2 have their posterior ends preserved. Two similar bones are arranged parallel to the left dentary and coronoid 2. The presence of a large tooth in cross-section (with plicidentine) permits the lower element to be diagnosed as a right dentary fragment, suggesting that the other bone is the right coronoid 2. Dermal bones of the posterior skull can be observed by reassembling all the blocks into the original concretion. Part of an opercle and other still unidentified bones are preserved.

**Remarks—**The distinctive large teeth on the dentary, the tiny teeth on the coronoid 2 and the presence of plicidentine visible in cross-section in MUHNCAL.20164, are conserved traits of Lepisosteidae (Grande, 2010). In addition, the gracile shape of the mandibular rami is also consistent with extant and fossil lepisosteids (Kammerer, Grande & Westneat, 2005). Prior to this research, the oldest known lepisosteid was represented by *Nhanulepisosteus mexicanus*, recovered from Kimmeridgian units of Mexico (Brito, Alvarado-Ortega & Meunier, 2017). The latter taxon extended the previously known chronostratigraphic range of Lepisosteidae 57 million-years back into the Upper Jurassic (Kimmeridgian). The current record from northern Chile additionally extends that range for almost 5 million years into the Oxfordian. MUHNCAL.20164 also represents the first lepisosteid found in the Jurassic of Gondwana, thus, representing a novel taxon. Previous lepisosteid fossil records from South America are known from the Lower Cretaceous of Brazil (Wenz & Brito, 1992; Brito et al., 2016), the Upper Cretaceous of Bolivia (de Muizon et al., 1983) and the Upper Cretaceous of Argentina (Cione, 1987). Other records from Gondwana are known from the Upper Cretaceous of Madagascar (Gottfried & Krause, 1998) and India (Rana & Kumar, 1990). MUHNCAL.20164 is here preliminarily described as part of this assemblage; however, it is currently under study and will be described in detail in a forthcoming contribution.

LEPIDOTIDAE Owen, 1860

Genus *SCHEENSTIA* López-Arbarello & Sferco, 2011

*SCHEENSTIA* SP.

(Figs. 6D–6G)

*Lepidotus*: In Biese, 1961

*Lepidotes* sp.: In Ossa-Fuentes et al., 2015

**Type species—***Scheenstia zappi* López-Arbarello & Sferco, 2011. Upper Kimmeridgian of Germany.

**Material—**MUHNCAL.20011, a postcranial articulated set of body scales lacking its caudal fin and its ventral margin. Cerro Campamento, west Calama. Cerro Campamento Formation, middle-to-upper Oxfordian. MUHNCAL.20194, a left mandibular ramus. Informal locality Biese 3, 400 m south of Loa River, west Calama. Cerro Campamento Formation, lower Oxfordian.

**Description—**MUHNCAL.20011 is preserved as a two-piece slab with the body exposed in left lateral view (Fig. 6D). The anterior half of the body shows scales in anatomical position, while the posterior half has slightly scattered scales. The dorsal outline of the body is well-preserved. The proximal part of the dorsal fin is preserved, with few parts remaining in their anatomical position. Nine or ten bones are preserved in the dorsal fin. Its preservation is poor, however, they likely belong to the dorsal fin rays instead to basal or fringing fulcra. Anterior to the dorsal fin, the postcranial margin is filled with an indeterminate number of dorsally-ridged scales, few of them with a spine shape. Body scales are rhomboid. Few of them permit observation of the peg-and-socket articulation, particularly in the few scales in the middle part of the preserved body. The presence of scale ridges is hard to assess due to the preservation. The midline is difficult to distinguish due to preservation. The dorsal outline is not softly convex, showing a marked inflection at both sides of the dorsal fin.

MUHNCAL.20194 is an almost complete left mandibular ramus, preserving twenty-four teeth. Lingually (Figs. 6E, 6F), the bone texture of the ramus appears as a spongy tissue, interpreted as prearticular-coronoid bone + dentine (Leuzinger et al., 2019). Labially (Fig 6H), the dentary shows a compact surface which is partially broken, likely during burial. The ramus has a high coronoid process and a massive overall shape with a deep anterior end, suggesting a deep symphysis. The posteroventral dentary process is absent. The teeth have hemispherical crowns with soft but worn enamel (no papillae were observed). The larger teeth occur in the internal (lingual) part of the ramus, while the smallest teeth occur in the labial perimeter. These have comparatively high basal attachment necks, while the larger interior teeth are strongly attached, without a neck. A single medial tooth is broken, which allows to observe a large pulp cavity and a thin enamel+dentine layer (Fig. 6G). Due to preservation, the latter features are hard to distinguish between, at first sight. In lingual view, three semi-open bony cavities are consistently placed under the three larger functional teeth.

**Remarks—**The body shape of MUHNCAL.20011 is commonly seen in Lepisosteiformes such as *Scheenstia* (López-Arbarello & Sferco, 2011: fig. 3), *Isanichthys* (Cavin & Suteethorn, 2006: fig. 1) and *Neosemionotus* (López-Arbarello & Codorniú, 2007: fig. 2). The dorsally inflected margin of the body is present indeed in the Barremian-Aptian *Scheenstia bernissartensis* (Cavin, Deesri & Olive, 2019: fig. 2E), the Tithonian *Scheenstia maximus* (López-Arbarello, 2012: fig. 21) and in the lower Tithonian *Scheenstia decoratus* (López-Arbarello, 2012: fig. 22), but is softly convex in the Upper Jurassic Lower Cretaceous *Isanichthys palustris* (Cavin & Suteethorn, 2006: fig. 1), *Isanichthys lertboosi* (Deesri et al., 2014: fig. 2) and in *Neosemionotus puntanus* (López-Arbarello & Codorniú, 2007: figs. 2 and 3). In addition, the presence of dorsally-ridged scales anterior to the dorsal fin is present in few species of *Scheenstia* (López-Arbarello, 2012: figs. 21 and 22) but absent in the upper Kimmeridgian *Scheenstia zappi* (López-Arbarello & Sferco, 2011: fig. 3). Under these considerations, MUHNCAL.20011 is referred to the genus *Scheenstia*, precluding a specific referral due to the incompleteness of the specimen.

MUHNCAL.20194 has a strongly tritoral dentition and a deep jaw symphysis, both features described for several species within the genus *Scheenstia* (López-Arbarello, 2012). It also possesses semi-open bony cavities under the medial functional teeth, with a high coronoid process, as described by Leuzinger et al. (2019) for a specimen referred to as *Scheenstia* sp. Based on these features, MUHNCAL.20194 is similarly referred to *Scheenstia* sp.

The Oxfordian age of MUHNCAL.20011 and MUHNCAL.20194 represents an extension for the *Scheenstia* biochron into the lower part of the Late Jurassic. Previously, *Scheenstia* was restricted to the Kimmeridgian-Aptian (López-Arbarello, 2012: fig. 25). The new records from northern Chile may represent the oldest known occurrences of the genus *Scheenstia*.

## Discussion

**Prior known record of Jurassic Actinopterygians from Chile**—The Lower Jurassic local record includes Sinemurian indeterminate Pycnodontiformes (Arratia & Cione, 1996), pachycormiform-like forms (Arratia & Cione, 1996) and indeterminate proleptolepids (Arratia, 1987; Arratia & Cione, 1996). An endemic whiteiid, *Atacamaia solitaria*, was also recorded in Lower Jurassic beds of northern Chile (Arratia & Schultze, 2015). During the Middle Jurassic, there are records in the Callovian of Cerritos Bayos (the same are as this study), with material referred to ‘*Lepidotes*’ and ‘*Pachycormus*’ (Biese, 1961). The latter were not figured and their repositories are currently unknown, rendering it impossible to reassess them. The Upper Jurassic record of northern Chile is exceptionally diverse and well-documented, including material of Oxfordian age referred to ‘*Lepidotes*’ sp. (Biese, 1961; Arratia & Cione, 1996; Ossa-Fuentes et al., 2015), *‘Pholidophorus*’ *domeykanus* (Arratia, Chang & Chong, 1975c; Arratia, 1987; Arratia & Schultze, 1999), *Protoclupea chilensis* (Arratia, Chang & Chong, 1975b), *Bobbichthys opercularis* (Arratia, Chang & Chong, 1975a), *Varasichthys ariasi* (Arratia, 1981), *Chongichthys dentatus* (Arratia, 1982), *Domeykos profetaensis*, *Protoclupea atacamensis* (Arratia & Schultze, 1985), *Antofagastaichthys mandibularis* (Arratia, 1986), *Atacamichthys greeni* (Arratia & Schultze, 1987), *Gyrodus* sp. (Martill, Chong & Pardo, 1998; Kriwet, 2001), and *Leedsichthys* sp. (Arratia & Schultze, 1999; Martill et al., 1999; Liston, 2007, 2008, 2010; Liston et al., 2013; Ossa-Fuentes et al., 2015). Kimmeridgian records include *Protoclupea* sp. (Arratia, 1987), previously described as ‘*Trissops*’ (=*Thrissops*) by Biese (1961), two taxa of indeterminate teleosteans (Arratia, 1987). Indeterminate actinopterygians and indeterminate pachyrhizodontoids have been described from Tithonian beds of central Chile (Arratia & Schultze, 1999). In the Jurassic/Cretaceous boundary at La Carreta, northern Chile, abundant remains of ‘semionotiforms’ (=Lepisosteiformes after López-Arbarello, 2012) referred to ‘*Lepidotes*’ (more likely, *Scheenstia*), have been mentioned (but not figured) by Arratia & Schultze (1999).

The presence of Amiiformes in Cerritos Bayos (the locality studied here) was mentioned by Casamiquela (1970), who commented on the local records referred to by Biese (1961) as ‘*Pachycormus*’ (misspelled as *Pachycornius* in Biese, 1961) to be within the clade Amiiformes. However, no descriptions or figures were provided, making verification problematic. The presence of indeterminate amioids in the Upper Jurassic of Chile was later mentioned by Arratia (1982) based on material recovered from the Oxfordian of Quebrada El Profeta, ca. 250 km south of Cerritos Bayos, although no description or detail of the material was provided. Later, Arratia (1986) indicated the presence of Amiiformes awaiting description, recovered from the same locality and age (there is no clarity as to whether this is the same material as mentioned in Arratia, 1986). Later, Arratia (1987) described material from the same locality (Quebrada El Profeta, Oxfordian) referring it to as indeterminate, ‘caturid-like’ amiiforms. The debated stem-teleost taxon *Atacamichthys greeni* Arratia & Schultze, 1987, from the Oxfordian of northern Chile, was recently considered to be likely related to Amiiformes due to its pectoral girdle features, their amioid scales and diplospondyl caudal vertebrae; however, this taxon also presents typical teleostean features such as the absence of coronoids and two hypohyals (Arratia, 2015).

**Lepisosteid records during the Upper Jurassic—**As defined by López-Arbarello (2012), Lepisosteoidea is the clade including all taxa more closely related to *Obaichthys* (clade Obaichthyidae) or to *Lepisosteus* (clade Lepisosteidae) than to *Pliodetes* or *Lepidotes*. The fossil record of obaichthyids is restricted to the Lower Cretaceous of Brazil and Morocco (Brito, Alvarado-Ortega & Meunier, 2017). Lepisosteids have been tracked back to the Lower Cretaceous, with records in the Aptian-Albian of Oklahoma, U.S. (Cifelli et al., 1997; Grande, 2010). More recently, the biochron of the Lepisosteidae was radically extended into the Upper Jurassic, with the record of *Nhanulepisosteus mexicanus* from the Kimmeridgian of Mexico (Brito, Alvarado-Ortega & Meunier, 2017). In this sense, considering the growing body of evidence regarding an Upper Jurassic marine vertebrate faunal interchange between the northern Tethys and southeastern Panthalassa (Arratia, 1994, 2008, 2015; Gasparini, 1996, 2009; Gasparini & Iturralde-Vinent, 2001; Iturralde-Vinent, 2003; Otero et al., 2020a, 2020b; Alarcón-Muñoz et al., 2021), the presence of a lepisosteid in the Oxfordian of northern Chile is chronostratigraphically and biogeographically sound. MUHNCAL.20164 represents the oldest member of the Lepisosteidae from Gondwana known to date.

**Mesozoic austral records of Pachycormidae—**The material studied here provides evidence of a pachycormid diversity in the Upper Jurassic of northern Chile, including macropredatory specimens with affinities to the genus *Hypsocormus*, as well as suspension-feeding specimens referrable to the gigantic genus *Leedsichthys*. Pachycormids from the Oxfordian Quebrada El Profeta have also been reported; a specimen referred to as *Hypsocormus*-like pachycormid was described by Arratia (1987: plate V), as well as specimens referred to *Leedsichthys* from the Quebrada San Pedro, Quebrada Aquada Chica, Quebrada Corral and Quebrada del Profeta localities (Liston, 2007, 2010). The clade Pachycormidae has been regarded as a rare group in the Mesozoic of Gondwana, with previous records mostly concentrated in Europe (Friedman et al., 2010; Maxwell et al., 2020). However, several records demonstrate the widespread presence of pachycormids in the austral Middle and Upper Jurassic (Arratia, 1987; Arratia & Cione, 1996; Martill et al., 1999; Gouiric-Cavalli & Cione, 2015a, 2015b; Gouiric-Cavalli & Arratia, 2022) even including Antarctica (Gouiric-Cavalli et al., 2019). Moreover, austral pachycormids have been recorded in the Lower Cretaceous (Kear, 2007) and in the Upper Cretaceous of former Gondwanic coasts (Cione et al., 2018; Otero & Suárez, 2021). Their discovery in the Oxfordian beds of northern Chile represents the oldest records of the group from Gondwana.

**Remarks on the local presence of ‘*Lepidotes*’—**This research confirms the presence of the historical taxon concept ‘*Lepidotes*’, profusely mentioned in previous works (Biese, 1961; Chong & Gasparini, 1976; Arratia, 1987; Arratia & Cione, 1996; Arratia & Schultze, 1999) but mostly lacking both adequate illustrations and known repositories (except by Arratia, 1987: plate IV; Arratia & Cione, 1996). The occurrence of this form is here demonstrated with formally housed material, being described and taxonomically reassessed within the genus *Scheenstia*, pending the specific description of the Chilean forms until more complete material has become available.

**Palaeoecology—**The marine environment of the studied unit (Cerro Campamento Formation) is indicated by the abundant presence of ammonoids, bivalves, gastropods and calcareous tubeworms (Biese, 1961), from which Baeza (1976) inferred a shallow, temperate-to-warm water environment, with reduced depth and enhanced water circulation. Charrier, Pinto & Rodriguez (2007) considered the Cerro Campamento Formation (former Campamento Member of the ex-Cerritos Bayos Formation) to represent a Late Jurassic regressive regime gradually passing into shallower facies, with evaporitic deposition (gypsum) and finely-laminated limestones in the upper section.

The assemblage collected from the Cerro Campamento locality was found mostly *ex-situ* from their stratigraphic occurrence but showed signs of limited transportation. This prevents the confident determination of a precise stratigraphic provenance, although the section exposed at Cerro Campamento belongs to the upper part of the unit, thus has a middle-to-upper Oxfordian age. The assemblage includes macropredators (mid-sized indeterminate Pachycormiformes, aff. *Hypsocormus* sp.), suspension-feeders (*Leedsichthys* sp.), and durophagous individuals (*Scheenstia* sp.). In addition, coastal vertebrates such as rhamphorhynchine pterosaurs have been found in levels of middle Oxfordian age (Alarcón-Muñoz et al., 2021). Except for the large-sized *Leedsichthys*, this diversity is consistent with the late regression of the basin pointed out by Charrier, Pinto & Rodriguez (2007), becoming a shallow coastal environment during the middle Oxfordian onwards. The presence of *Leedsichthys* remains in this environment could be explained by strandings in shallower waters and/or by carcasses that reached coastal waters, as similarly documented among extant and Neogene balaenopterid whales (Pyenson et al., 2014; Fretwell et al., 2019).

On the other hand, the diversity found in the lower Oxfordian levels at the Biese 3 informal locality includes macropredators (Lepisosteidae indet.) and durophagous individuals (*Scheenstia* sp.), occurring in association to larger diapsids such as pliosaurids, cryptoclidids, ophthalmosaurids and metriorhynchids (Soto-Acuña et al., 2015, 2021; Otero et al., 2018, 2020a, 2020b), as well as remarkably abundant ammonoid phragmocones (Rodrigo A. Otero, 2021, personal observations), suggesting comparatively deeper waters.

**Comparison with local coeval Actinopterygian assemblages—**The remarkable actinopterygian diversity from the Oxfordian levels of the El Profeta Formation in northern Chile (ca. 250 km south from the outcrops of the Cerro Campamento Formation) is represented by Crossognathiformes that include most members of the clade Varasichthyidae, among them, *Protoclupea chilensis* Arratia, Chang & Chong, 1975b, *Bobbichthys opercularis* (Arratia, Chang & Chong, 1975a), *Varasichthys ariasi* Arratia, 1981, *Domeykos profetaensis* Arratia & Schultze, 1985 and *Protoclupea atacamensis* Arratia & Schultze, 1985. In the same unit, additional Crossognathiformes includes the monotypic clade Chongichthyidae, represented by *Chongichthys dentatus* Arratia, 1982. The same unit has yielded the *incertae sedis* teleostean taxa *‘Pholidophorus*’ *domeykanus* (Arratia, Chang & Chong, 1975c), *Antofagastaichthys mandibularis* Arratia, 1986, *Atacamichthys greeni* Arratia & Schultze, 1987, and specimens referable to *Gyrodus* sp. (Martill, Chong & Pardo, 1998; Kriwet, 2001), *Leedsichthys* (Arratia & Schultze, 1999), indeterminate ‘Semionotiformes’ and indeterminate, ‘?*Hypsocormus*-like’ macropredatory Pachycormiformes (Arratia, 1987).

On the other hand, the actinopterygian fauna from the Cerro Campamento Formation shows similar coeval forms to as those reported from the El Profeta Formation, with the presence of pachycormiforms (Pachycormidae) including both macropredators and suspension-feeders, as well as the record of *Protoclupea* sp. (Arratia, 1987), a genus previously reported with two species in the El Profeta Formation (Arratia, Chang & Chong, 1975b; Arratia & Schultze, 1985). Lepidotids have been reported in both the Oxfordian El Profeta Formation (Arratia, 1987) and the Oxfordian Cerro Campamento Formation, the latter with material first referred to ‘*Lepidotes*’ sp. (Ossa-Fuentes et al., 2015) and here reassessed as *Scheenstia* sp., with description of additional material (MUHNCAL.20194). In contrast, novel records from the Oxfordian Cerro Campamento Formation include the presence of a marine, still indeterminate Lepisosteidae. In addition to these differences, the record of coeval Crossognathiformes and stem-telosts commonly present in the El Profeta Formation (*i.e*., *Varasichthys*, *Protoclupea* spp.; *Chongichthys, Domeykos*, *Atacamichthys, Antofagastaichthys*) seem to be scarce in the Cerro Campamento Formation (so far only represented by a single Kimmeridgian record of *Protoclupea* sp.; Arratia, 1987). The reasons for this bias are still unknown, possibly being related to environment and paleogeography.

The Upper Jurassic vertebrate diversity of the Cerro Campamento Formation still lacks a few elements typically found in other coeval localities. As an example, there is a significant absence of chondrichthyans, a group which is well-represented in northern Tethys during that time period (see Kriwet & Klug, 2004 and references therein). In contrast, Upper Jurassic chondrichthyans from southeastern Panthalassa are sparsely represented by two discoveries restricted to the Tithonian of central Chile, including an isolated tooth of an indeterminate Synechodontiformes (Suárez & Otero, 2011) and dental plates referable to the chimaeriform genus *Ischyodus* (Otero et al., 2021). The local absence of chondrichthyans is currently difficult to explain, considering that the environment of the Upper Jurassic northern Chile seems to have been propitious for these organisms, especially considering the demonstrated marine vertebrate interchange with northern Tethys *via* the Hispanic, Viking and Mozambique corridors (Gouiric-Cavalli, 2017). Furthermore, chondrichthyans are extremely rare in the austral Lower Cretaceous and these scarce specimens remain unstudied (Bell & Suárez, 1997).

## Conclusions

This study presents new discoveries of ray-finned fishes from the Upper Jurassic of the Atacama Desert in northern Chile. The material was collected from two localities in the Cerro Campamento Formation, southwest Calama, Región de Antofagasta, Chile. A first assemblage, of middle-to-upper Oxfordian age, is composed of macropredatory, indeterminate Pachycormiformes, pachycormids with affinities to the genus *Hypsocormus*, suspension-feeding pachycormids referable to *Leedsichthys* sp., and lepidotids referable to *Scheenstia* sp. The second assemblage, of lower Oxfordian age, consists of a more discrete diversity that includes *Scheenstia* sp., as well as a still indeterminate lepisosteid skull. This study sheds light on the ray-finned diversity from the Upper Jurassic of southeastern Panthalassa, previously dominated by Crossognathiformes (*e.g*., Varasichthyidae, Chongichthyidae). It also validates the previously suggested presence of macropredatory pachycormids. Lepidotids have been frequently mentioned as being present in the Upper Jurassic of northern Chile, but rarely figured or described. This study provides new material of both lower Oxfordian and middle-to-upper Oxfordian age, demonstrating the continuous presence of lepidotids in northern Chile during this period, and updating its taxonomic status to the genus *Scheenstia*, in consideration of the anatomical features and chronostratigraphic occurrence of the Chilean material.

With the exception of a single Kimmeridgian record of *Protoclupea* sp. in the study area, it must be noted that the assemblages from the Cerro Campamento Formation are characterized by a general absence of typical Crossognathiformes such as those described in the El Profeta Formation exposed ca. 250 km south from the studied localities. The reasons for this bias are currenly poorly understood, possibly being related to environmental differences (shelf depth), geographical barriers, and/or the local declination of each group during the Oxfordian-Kimmeridgian.

## Institutional Abbreviations

MUHNCAL: Museo de Historia Natural y Cultural del Desierto de Atacama, Calama, Chile
NHMUK: Natural History Museum, London, UK
SMNK: Staatliches Museum für Naturkunde, Karlsruhe, Germany

## Additional Information and Declarations

### Field Study Permissions

The following information was supplied relating to field study approvals (*i.e*., approving body and any reference numbers): Retrieval of paleontological specimens in Chilean territory was granted by the Consejo de Monumentos Nacionales (National Monuments Council) in Ord. 1464/2018.

### Data Availability

The following information was supplied regarding data availability: This research studied the fossil specimens MUHNCAL.20006, MUHNCAL.20011, MUHNCAL.20039, MUHNCAL.20148, MUHNCAL.20150, MUHNCAL.20164, MUHNCAL.20193 and MUHNCAL.20194, which are housed in the Museo de Historia Natural y Cultural del Desierto de Atacama, Calama, Chile.
